# Driving Cells with Light‐Controlled Topographies

**DOI:** 10.1002/advs.201801826

**Published:** 2019-05-20

**Authors:** Alberto Puliafito, Serena Ricciardi, Federica Pirani, Viktorie Čermochová, Luca Boarino, Natascia De Leo, Luca Primo, Emiliano Descrovi

**Affiliations:** ^1^ Candiolo Cancer Institute FPO‐IRCCS Candiolo Turin 10060 Italy; ^2^ Department of Oncology University of Turin Turin 10060 Italy; ^3^ Department of Applied Science and Technology Polytechnic University of Turin C.so Duca degli Abruzzi 24 Turin 10129 Italy; ^4^ Department of Chemical Engineering University of Chemical Technology Prague Technická 3166 28 Praha 6 Czech Republic; ^5^ Quantum Research Labs & Nanofacility Piemonte Nanoscience & Materials Division Istituto Nazionale di Ricerca Metrologica Strada delle Cacce 91 Turin 10135 Italy

**Keywords:** cell‐instructive substrates, cell migration, cell orientation, light‐responsive polymers, optical manipulation

## Abstract

Cell–substrate interactions can modulate cellular behaviors in a variety of biological contexts, including development and disease. Light‐responsive materials have been recently proposed to engineer active substrates with programmable topographies directing cell adhesion, migration, and differentiation. However, current approaches are affected by either fabrication complexity, limitations in the extent of mechanical stimuli, lack of full spatio‐temporal control, or ease of use. Here, a platform exploiting light to plastically deform micropatterned polymeric substrates is presented. Topographic changes with remarkable relief depths in the micron range are induced in parallel, by illuminating the sample at once, without using raster scanners. In few tens of seconds, complex topographies are instructed on demand, with arbitrary spatial distributions over a wide range of spatial and temporal scales. Proof‐of‐concept data on breast cancer cells and normal kidney epithelial cells are presented. Both cell types adhere and proliferate on substrates without appreciable cell damage upon light‐induced substrate deformations. User‐provided mechanical stimulation aligns and guides cancer cells along the local deformation direction and constrains epithelial colony growth by biasing cell division orientation. This approach is easy to implement on general‐purpose optical microscopy systems and suitable for use in cell biology in a wide variety of applications.

Mechanotransduction, i.e., the transformation of mechanical stimuli into biochemical signaling, has been the subject of numerous studies in the last two decades, due to its importance in a wide variety of contexts ranging from embryonic development and regeneration to homeostasis and cancer progression.^1^, ^2^, ^3^, ^4^ Indeed, mechanical stimuli are at the basis of several fundamental biological mechanisms, such as growth control, migration and invasion, cell differentiation, and tissue‐level organization.

Cells are mechanically influenced by the substrate both as a consequence of its molecular composition and of its larger scale structure, such as the presence of long filamentous structure, the presence of curvature, or the variation in density and rigidity.^5^ An extremely powerful experimental technique to exploit mechano‐sensing to influence cell behavior is the engineering of substrate topographies.^6^, ^7^ Such approach consists in seeding cells on synthetically fabricated substrates that present ridges or valleys of different size and shape that are able to influence the cell. The most commonly used techniques to fabricate micro and nanometric patterns are photo‐ and soft lithography and micromachining and can be used to create topographical features on a wide variety of scales. The presence of such patterns at both micro and nanoscale has been shown to influence cytoskeletal reorganization, focal adhesion dynamics, and cell contractility (see, e.g., refs. [8,9], and references therein). The proper engineering of a substrate has been shown to modify cell differentiation as well.^10^, ^11^, ^12^, ^13^ Therefore micro and nanofabrication of substrates is a powerful tool to explore the mechanisms of cell migration and to study the relation between mechano‐sensation and that of the corresponding cell migratory activity from biochemical to macroscopic level.

Different geometries have been shown to promote cell orientation and migration along ridges 14, 15 and cell differentiation.^10^, ^11^, ^12^ However, most of the micro/nanostructures available at present exhibit fixed morphologies that cannot be finely modified, once manufactured. Such a limitation is particularly relevant as it hampers the possibility to perform measurements on the time‐ and space‐varying effects of substrate morphology on cell adhesion, migration, and differentiation.

The substantial inability of conventional substrates to be mechanically tuned on demand can be overcome by using stimuli‐responsive polymers.^16^ These materials are able to respond to external stimuli such as environmental pH, temperature, acoustic waves, electric and magnetic fields, electromagnetic waves, and radiation by changing some of their physical/chemical properties, including morphology.

In this framework, light represents a very attractive external trigger, as it can be noninvasively provided according to arbitrary spatio‐temporal patterns at different wavelengths and polarization states.^17^ Light responsivity is typically obtained by dispersing or chemically binding photoactive units within the polymer network.^18^ Azobenzenes are among the most popular molecular photoswitches because of their ability to undergo a conformational change between two isomeric states when irradiated with UV or visible light.^19^, ^20^ Such a photoisomerization is crucial in triggering the so‐called directional photofluidization^21^, ^22^ and induce deformation effects in azopolymers,^23^, ^24^ which have been extensively exploited as a fabrication mean to reversibly inscribe/erase surface relieves (SR) on continuous, homogenous planar films.^25^, ^26^, ^27^, ^28^, ^29^

When dealing with 2D light‐responsive substrates, topographies can be instructed by a direct laser writing exposure of azopolymeric films previously spun on planar glass substrates.^30^ In this way, arbitrary geometries can be obtained by properly controlling the position of a scanning laser in a confocal microscope system.^31^, ^32^, ^33^ As an alternative, projection masks 34 or laser interference lithography can be used.^35^, ^36^, ^37^ Exposure with circularly polarized light or temperature ramps can quickly erase the inscribed topographic patterns,^36^, ^38^ thus allowing multiple writing/erasure steps on azopolymeric film hosting cells already attached on the surface.^32^, ^33^, ^39^ It should be observed, however, that the mechanism underlying the SR formation is affected by strong interactions with the substrate itself, thus limiting the maximum corrugation height (lower than 1 µm) produced on the film surface.^40^, ^41^ From the biological point of view, such constraints are known to limit the effect of topographies on cell orientation.^42^, ^43^ Moreover, the presence of a uniform residual layer of azopolymer remaining beneath the inscribed corrugations can cause adhesion and stability issues on the glass substrate.^44^

Here, we present an approach to spatio‐temporally control substrate topographies starting from a prepatterned azopolymeric structure on a glass‐bottom Petri dish. Topography modifications are performed nonsequentially in a modified inverted microscope, with a single exposure of the target area with a properly controlled laser distribution. A short (few tens of seconds) illumination of the as‐fabricated structure results in stable (months) and reproducible deformation patterns which can be customized according to the biological need. More specifically, depending on the local polarization orientation and intensity of the laser, micropillars get elongated along a direction parallel to the laser polarization, thus resulting in a generally both anisotropic and inhomogenous microtextured surface.

The topography modifications that can be obtained with the presented approach lie in the micrometer range, therefore allowing a particularly effective dynamical stimulation for biological purposes.

Importantly, due to the short irradiation times, micropillar deformation can be performed directly on substrates hosting living cells on‐board. We present an experimental validation of our technical approach by showing orientation and directional migration of cancer cells and growth confinement of epithelial colonies on deformed topographies. These two case studies were chosen as representative of classes of problems on single cell and multicellular scale that can be tackled by our technical approach. A discussion of several possible extensions of the proposed method is presented.

The deformation capabilities of azopolymeric microstructures under irradiation by moderate local laser intensity are known from several previously published works.^45^, ^46^, ^47^ The extent of the pillar cross‐section elongations is determined by the polarization direction of the illuminating light field, and can be made reversible as well depending on the chemical formulation and mechanical properties of the azopolymeric blend.^48^ The azopolymer is transferred onto a standard glass‐bottom Petri dish in the form of micropillar arrays that can be spatially arranged according to a given geometry, such as a square lattice. Micropillars can be arbitrarily sized and soft‐printed from a polydimethylsiloxane (PDMS) stamp manufactured according to conventional fabrication methods (see the Experimental Section).

In **Figure** **1**a, a sketch of the optical setup is shown, including a customized laser projection system coupled to an inverted microscope equipped for biological time‐lapse observations. A linearly polarized laser beam is spatially filtered, expanded, and collimated toward a programmable liquid crystal phase element (spatial light modulator, SLM), which is computer‐controlled. In this way, arbitrary intensity distributions are produced at the entrance of the microscope lateral port, by means of an external imaging stage. The polarization state of the laser pattern is varied by means of a motorized polarization element (including a half‐wave plate crystal) that can be operated in synchronization with the SLM. The microscope internal optics then projects the laser pattern onto the sample, according to the desired magnification selected by the objective. Upon laser projection, each micropillar elongates according to the local laser intensity and polarization direction.

**Figure 1:**
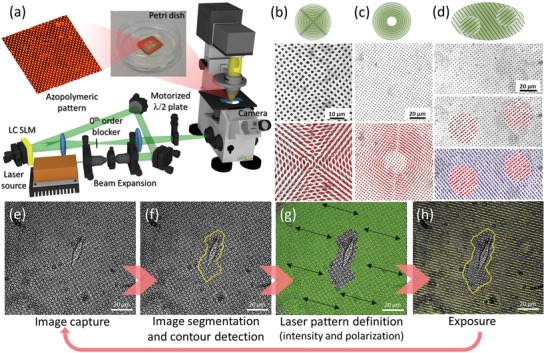
Platform concept. a) Laser projection system coupled to a commercial inverted microscope for life sciences. Two computer‐controlled elements are herein introduced: a phase‐only liquid crystal‐based SLM to dynamically generate arbitrary laser patterns to be projected onto the micropillar arrays, and a half‐wave plate mounted on a motorized rotational stage for rotating the laser linear polarization according to any desired orientation. Proper opto‐mechanics is employed for alignment purposes. b–d) Representative deformation patterns of an array of azopolymeric micropillars obtained upon irradiation with a laser pattern having hyperbolic polarization, b) with a 50 s exposure time, c) doughnut‐shaped azimuthal polarization projected, with a 90 s exposure time, and d) spatially dependent linear polarization states projected, with a 90 s exposure time. In the latter case, the pattern is obtained by superposing a time‐sequence of two complementary spatial patterns, each one having a defined linear polarization state as synchronously set by the motorized half‐wave plate. Objective magnification is 20×. e–h) Sequence of operations illustrating the conceptual work‐flow of a prototypical use of the proposed platform. e) First, an initial image of the sample is captured, wherein one or more target cells are included. Here, a single cell is imaged in bright field with a 20× objective. Then, a processing stage lets the user defining a proper mask to be used as an illumination pattern. f) In alternative, an automatic segmentation algorithm can identify the object(s) outline(s) and produce a corresponding binary mask. g) The binary mask is fed into the SLM, in order to produce a corresponding laser pattern (e.g., conformal to the target cell in the case shown here) with a desired polarization state which is ruling the deformation direction(s) on the pillars. Here, the polarization is uniform over the whole irradiated area and oriented as indicated by the black arrows. h) Finally, the deformation is produced on the micropillars and directly observed on the microscope camera, in live capturing (see Movie M5, Supporting Information). The laser exposure time can be either fixed a priori or defined by the user, depending on the specific application case. This work‐flow can be iterated at any time, and made adaptive to the specific evolution of the observed target cells distribution.

Representative deformation patterns are shown in Figure 1b–d, wherein bright‐field optical images before and after laser irradiation are presented. In Figure 1b, a laser field having a hyperbolic‐like polarization distribution is used. As a result, micropillars are elongated according to the local orientation of the polarization vector. In Figure 1c, a doughnut‐shaped laser beam with azimuthal polarization is employed. In this case, pillars located in the dark central region of the beam are left unchanged, while illuminated pillars elongate to form a set of almost continuous concentric rings (Movies M1–M3, Supporting Information). In Figure 1d, two complementary intensity patterns are sequentially projected, having mutually orthogonal linear polarizations (Movie M4, Supporting Information). The deformation of pillars over time is illustrated in Figure S4 in the Supporting Information. In all presented cases, the extent of elongation is such that contiguous micropillars are often close to merge together. As a result, an average anisotropic surface is obtained over supra‐micrometric lengths. A further level of control can be attained by many possible alternative micropillar arrangements (e.g., with a different spatial distribution, density gradients or nonuniform sizes, and high aspect ratios), the only limitation being the availability of a proper master for soft‐printing via PDMS stamps. Differently from other arrangements in which light is used to control topographic features on a nanometric scale,^44^ our approach allows lateral deformations of micropillars in the micrometer range, which is large as compared to the pillar cross‐section, but still much smaller than the typical dimension of cells attached thereon.

The capability to elongate micropillars along any desired direction within specific regions of the sample is exploited here in an adaptive fashion, with living cells attached on the substrate. To illustrate this we first seed MDA‐MB‐231 cells on poly‐DR1M micropillars (see the Experimental Section). Once attached to the pillar arrays, we identify one or more proper target cells to focus on. In the procedure illustrated as a flow in Figure 1e–h, we show how the pattern region surrounding a single target cell can be instructed, while leaving the cell unperturbed. In this way, the amount of direct irradiation to cells and the possible phototoxicity induced are minimized. Starting from a captured bright‐field image of the cell, a digital image representing a black/white or gray‐scale binary mask can be generated either by the user (with an external image manipulation software) or automatically (with a conventional contour recognition algorithm). The mask is processed and fed to the SLM (see the Experimental Section) after a proper size scaling of the mask depending on the objective magnification used for projection. The linear polarization of the laser is rotated in order to match the desired deformation direction(s). Upon proper alignment of the laser wave‐front (see the Experimental Section), a laser pattern complementary to the target cell outline is projected onto the sample surface (exposure time 140 s), thus triggering the pillar deformation (see Movie M5, Supporting Information). After laser irradiation, the target cell can be monitored in time while responding to the deformed substrate. At a later time, such a procedure can be either iterated by taking into account the new cell location or repeated for other target cells previously identified. The advantage of a nonscanning, one‐shot exposure can be readily appreciated when broader areas are intended to be addressed, by using low‐magnification objectives, possibly at the cost of a decreased projected laser intensity and/or a longer exposure time. With the setup described in the Experimental Section, 10× objective, 4 min exposure, 20 mW laser power, deformation of a substrate region including several cells did not induce a noticeable phototoxic damage, as shown in Movie M6 in the Supporting Information. Further data of pillar deformations with living cells on‐board and on the induced phototoxicity are presented in Figures S4, S5, and Movie M14 in the Supporting Information and no significant toxicity is observed. We conclude that these experimental conditions are completely compliant with most cell biological assays.

As a first application of the proposed approach, we decided to test the ability of cancer cells to respond to substrate mechanical stimulations. Cancer cells can acquire different capabilities to better survive in hostile environments (such as the metastatic niche, e.g.). The degree of sensitivity of cancer cells to external mechanical cues and their ability to fit a nonnative microenvironment are related to the malignancy of the cells. With this idea in mind, we selected the highly aggressive breast cancer cell line MDA‐MB‐231, originating from a triple‐negative breast tumor, as a good candidate to test our approach.^50^

We seeded MDA‐MB‐231 on patterned substrates and we observed the expected degree of cell adhesion and proliferation, comparable to tissue culture plasticware. We therefore assessed whether such cells would be sensitive to substrate deformation, despite their extremely aggressive phenotype.

After at least 24 h from seeding, substrates were laser‐illuminated and correspondingly deformed in selected regions with various patterns and after other 24 h cells were fixed and decorated in order to see actin and nuclei as shown in **Figure** **2**a,b (see the Experimental Section for details). Such stainings allow identification of cells and are suitable to measure the degree of alignment along the local direction of deformation. The degree of cell alignment was measured by segmenting cell outlines and using shape and geometrical features as a proxy for orientation and polarization (Figure 2c). The degree of orientation with respect to the local direction of the deformed pattern for each cell was quantified for several deformation patterns, as shown in Figure 2d. The orientation of the vast majority of cells on deformed substrates is qualitatively appreciable. In statistical terms, the median of the angle distribution on deformed substrate is 22.4°, therefore for half of the cells, the deviation with respect to the direction of deformation is at most within 20°, as shown in Figure 2d.

**Figure 2:**
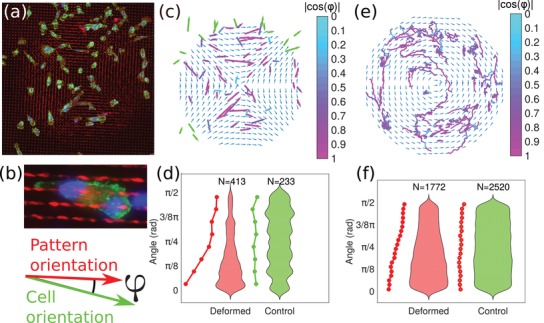
Adhesion and migration of cancer cells on deformed substrates. a) Snapshots of fixed MDA‐MB‐231 seeded on deformed substrates, stained for polymerized actin (phalloidin, green), nuclei (dapi, blue or DRAQ5, red), and pillars (auto‐fluorescence, red). Cells are oriented along the direction of deformation of the substrate and show aligned stress fibers. b) Sketch of the conventional directions used in the figure. The angle φ between the local direction of the deformed pattern and the direction of the major axis of the cell is used as a measure of alignment. c) Quantification of cell polarization and orientation on deformed substrates corresponding to panel (a) (with local angle indicated by blue arrows). Each cell is depicted here as an arrow which length is proportional to the ratio between major and minor axis lengths, and which orientation is that of the major axis. Green arrows indicate cells sitting outside the deformed region, i.e., undeformed pattern, while arrows colored from blue to magenta indicate cells sitting onto the deformed region. The color‐map indicates the absolute value of the cosine of the angle φ. Alignment of cells is qualitatively clear. d) Quantification of cell alignment over deformed versus undeformed substrate shown as a violin plot. Continuous lines represent the probability distribution of the angle. Kolmogorov–Smirnov 49 statistical test yields rejection of the null hypothesis with a *p*‐value of 5.910^−14^. e) Tracks of cells migrating on deformed substrates are shown to illustrate directed migration. The orientation of cell displacements in each trajectories (corresponding to segments of 1 h) was projected onto the local orientation of the pattern by calculating the cosine of the angle φ. The color‐map indicates the degree of alignment. The deformation pattern is shown by blue arrows while each line is a cell trajectory. f) Statistical distribution of displacement orientation on deformed versus undeformed patterns shown as a violin plot. Continuous lines represent the distribution of the angle. Kolmogorov–Smirnov statistical test yields rejection of the null hypothesis with a *p*‐value of 6.910^−185^.

To further understand the effect of the instructed topography on cells, we assessed the degree of cell polarization, i.e., cell elongation, on a deformed substrate against that on undeformed substrates. Cell polarization is measured here as a major to minor axis lengths ratio. It is worth noting that cell elongation on nondeformed substrates is the relevant control for our experiment as opposed to cells seeded on flat nondeformed substrates. This is due to the fact that a flat substrate offers a continuous surface while a pillar‐based substrate only has discretely distributed adhesion points. We compared the distribution of cell polarization (i.e., the distribution of the aspect ratio) and found only minor variations of cell polarization distribution in the two samples. This has four consequences: i) cells are able to deform and adhere on the substrate irrespective of the deformation; ii) cell orientation upon substrate deformation is not caused by a mere increase in cell polarization (see Figure S3, Supporting Information); iii) to observe a statistically significant mean elongation difference one can design pillars with different pitch, fill factor, and depth, while seeding cells at lower densities in order to have isolated cells; and iv) curvilinear deformations with curvature radii comparable to cell lengths are not effective at inducing maximum elongations (as expected).

Next, we investigated the induction and persistence of alignment overtime, by measuring cell migration along the direction of the deformation. To this aim, we performed time‐lapse microscopy on cells infected with viruses allowing visualization of nuclei by fluorescent proteins tagging. We seeded cells on undeformed substrates, waited for the cells to adhere and then deformed according to hyperbolic, azimuthal, and linear profiles. 24 h postdeformation, we monitored cells moving for about 3 days as shown in Movies M7–M10 in the Supporting Information. By performing cell tracking, we were able to record several single cell trajectories, and to correlate them with the deformation pattern instructed on the substrates. The results of this analysis are shown in Figure 2e, where alignment of the trajectories along the deformation direction is qualitatively clear. Statistical comparison of 1 h segments of all trajectories confirmed that indeed trajectories on deformed substrates are aligned along the deformation direction (see Figure 2f).

Our analysis demonstrates that mesenchymal‐like cancer cells adhere and elongate on azopolymeric microtextured patterns, regardless of the imposed deformation, indicating that neither patterning nor deformation alters the adhesion process appreciably. However, the local anisotropy induced on the deformed pattern is effective in orienting the cell bodies along the deformation direction. Moreover, time‐lapse analysis revealed that cells are viable and actively migrating on the substrate along a stable orientation as instructed by the laser polarization.

Epithelial cells, as opposed to typical cancer cells frequently showing mesenchymal features, tend to develop tight cell–cell junctions which introduce yet another mechanical input to cell behavior, i.e., that of intercellular interactions. Indeed, cultured epithelial cells grow attached to each other by means of specialized junctions strongly biasing cell movement and form colonies. In the bulk of large colonies, cells tend to move very little and to control cell proliferation tightly, a phenomenon commonly called contact inhibition,^51^ while closer to the boundary, cells tend to migrate outward, on average perpendicularly to the colony edge. The complexity of such a biological model makes the control of the colony growth by means of mechanical stimuli through the substrate topography particularly intriguing.

To verify whether we could influence epithelial growth by spatio‐temporally controlling substrate topography, we cultured a commonly used kidney epithelial cell line (Madin–Darby Canine Kidney, MDCK) on patterned substrates to be exposed with proper laser illumination. Differently from the previous set of experiments on cancer cells, in this case, colony‐wide regions exhibiting a homogenous anisotropy are required to be induced.

MDCK was able to attach and form colonies on all undeformed patterns as shown in **Figure** **3**a and Movie M11 in the Supporting Information. Division time was consistent with previously observed behaviors and estimated as roughly 26 h. Expansion of the colonies on undeformed substrates was qualitatively isotropic, with cells moving on average perpendicularly to the edge of the colony, as shown in Figure 3c,d. The general orientation of the colony (defined as the orientation of the major axis of the colony outline) had no preferential direction over time.

**Figure 3:**
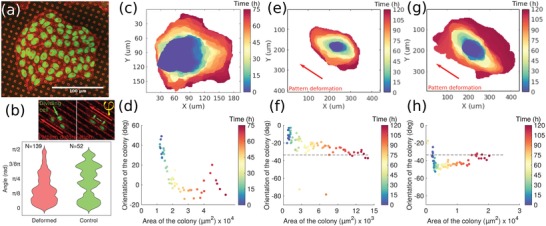
Substrate topography can bias the growth of epithelial colonies. a) Microscopy image showing LifeAct‐Ruby/H2B‐GFP MDCK cells cultured on patterned substrates. b) Quantification of cell division positioning with respect to deformation of the pattern. The top panel represents a dividing cell, and the definition of the deviation between the orientation of cell division (green arrow) and the orientation of the deformation (red arrow). The lower panel compares the distributions of angles on deformed versus undeformed patterns. Statistical significance was assessed by means of a Kolmogorov–Smirnov test, yielding rejection of the null hypothesis with a *p*‐value of 310^−3^. c,d) Colony outlines as a function of time showing time progression of the colony, alongside with corresponding quantification of the elongation and orientation on undeformed pattern. The outline was extracted by segmenting the LifeAct‐Ruby signal. e–h) Same as previous panels, except with linear deformation. For all these four panels, the orientation of the pattern is indicated by the dashed black line.

After inducing micropillars deformation along specific directions on regions surrounding growing cell colonies at an early stage, we measured the colony outlines as a function of time (over almost 3 days). The outline of the colony was in most cases oriented along the direction of deformation of the patterns, showing that topography can indeed influence epithelial cell growth, despite the presence of cells in the bulk moving apparently in all directions (Figure 3e–h and Movie M12, Supporting Information). Indeed cell migration was not evidently constrained by the presence of the deformed substrate and showed collective circular motion, as evident from Movie M12 in the Supporting Information. Since the observed polarized growth is found to be uncorrelated with cell migration, we drew our attention to possible anisotropies in cell division orientation. The latter is measured as the direction defined by the positions of the two daughter cells right after mitosis. On undeformed substrates, cell division was generally isotropic, while a significant anisotropy was observed on deformed patterns (Figure 3b and Movie M13, Supporting Information).

This unexpected result leads us to speculate about a possible role of the substrate morphology on spindle positioning and illustrates well the effectiveness of our technological approach and the potential impact on the field of cell biology.

We describe here a novel approach to exploit light‐responsive materials to trigger and measure biological activity in response to different custom topographies. We have shown that cancer cells, upon deformation of the substrate with proper spatially inhomogenous patterns, are able to sense the local deformation direction and orient and migrate accordingly. We have also applied our approach to the problem of controlling the growth of epithelial colonies and we have shown that deforming the patterned substrates induces growth to be preferentially in the direction of the deformation due to the orientation of cell division.

The presented experimental approach covers the stimulation of a biological response in both space and time, that importantly can be controlled independently. Spatial scales range from sub‐cellular to multicellular level. The lower bound to temporal scales is substantially determined by the light‐responsivity of the polymer blend and the extent of the desired induced anisotropy, provided the phototoxicity from laser irradiation is tolerated.

Small and fast scales can be reached thanks to the fast responsivity of the azopolymer and is greatly improved by two technical advantages of our approach: i) deformations occur at once and do not require laser scanning and ii) substrate stimulation and biological observation can be performed simultaneously. Such features allow to employ our setup to study biological phenomena that occur on the scale of a few tens of seconds and on the spatial scale of a cell, such as calcium signaling, e.g., which has been shown to be influenced by topographies.^52^

Large and slow scales can also be explored thanks to the possibility of obtaining stable (in our experiments, months) topography modifications over large areas, by means of irradiation with low‐magnification lenses. Such is the case of some of the phenomena explored here, like epithelial growth. Of note, another extremely interesting scenario can be faced: that of temporally fast and spatially large scales. This situation is the most extreme and gives access to a number of collective phenomena in biology, that occur on temporal scales of a few minutes, or even less than a minute, and on spatial scale of several hundreds of microns. Phenomena like these have been shown, e.g., in protein activation waves^53^, ^54^ or yet in multicellular calcium waves.^55^, ^56^ Unfortunately, the applicability of methods like the one presented in the current manuscript is currently limited to those biological problems where interactions with the substrate are relevant. In such cases, it is however crucial to control the mechanical stimulus over a large area in a relatively small time, in order to be able to directly correlate the occurrence of biological phenomena to the deformation event.

Some aspects of the proposed technique can be modified or expanded in order to be more suitably adapted to specific biological needs. For example, alternative compositions of the light‐responsive polymeric substrate can be used, wherein mechanical properties such as elasticity or deformation reversibility are enhanced (e.g., as for azobenzene‐doped elastomers^57^). Deformable materials could be exploited to study the effects of stretching or compression^58^, ^59^ on cell growth, revealing the impact of local (single cell scale) or global (tissue level scale) mechanical deformation of the substrate. Reversibly deformable substrates could also be employed to simulate the cyclic stretch for stimulation and conditioning of cardiomyocites and for regenerative purposes.^60^, ^61^

We have chosen to employ micropillars as this particular geometry allows significant deformation to be reached and this has been successfully used in the past to show important cell biology concepts.^62^ However, our approach can be generalized to other fabrication geometries, such as hexagonal patterns, random distributions, or patterns with variable density of features. Such a choice would allow to study other biological phenomena such as, e.g., rigidity‐guided movement (durotaxis) and surface‐attached chemicals‐driven movement (haptotaxis).

A relevant generalization to this approach is the optical triggering of 3D scaffolds or 3D matrices that are still amenable to light‐driven modifications.^63^ Indeed, with the advent of 3D cultures and, more recently, the wide diffusion of organoids as model system, the need of having an experimental tool‐set to probe mechanical effects on cell aggregates exerted through the matrix is growing. Azobenzene‐containing liquid crystalline networks (LCN)^64^, ^65^, ^66^ constitute potentially attractive materials to manufacture light‐responsive, bio‐compatible substrates for cell cultures.^67^ Beside the inherent capability to induce a unidirectional cell alignment according to the LC molecular configuration (e.g., the orientation of the nematic director in flat films such as in ref. [68]), light‐responsive LCN can be prepatterned and employed to tune cell adhesion and migration via a dynamic control of the nanoscale roughness.^69^ In recent works, bio‐compatible LCN films have been demonstrated to exhibit reversible local morphological changes of about 100 nm in height, with a lateral resolution of roughly 10 µm, upon moderate irradiations.^70^, ^71^ Conversely, our approach can be advantageously employed to provide multiple complex spatio‐temporal light‐stimulation patterns over large‐surface scales, with micrometric lateral resolution and height, e.g., by exploiting the fast reconfigurability of SLM‐based projection systems on micropatterned surfaces.

In conclusion, the approach presented here proposes a technical implementation that is broadly accessible to researchers with diverse backgrounds and features a number of technical solutions that make it suitable for the study of a wide variety of biological problems.

## Experimental Section

*Sample Fabrication*: Poly(disperse red 1 methacrylate), pDR1M (Sigma‐Aldrich) azopolymer was employed to fabricate light‐responsive 2D pillar arrays by soft‐printing technique. Poly‐DR1M was one of the most well‐known and largely used azopolymers in SR fabrication and easily available in the market. pDR1M was first dissolved at 2 wt% in *N*,*N*‐dimethylformamide and then drop‐casted onto a Petri dish (glass, #1.5 cover‐slip). A PDMS stamp previously obtained from a laser‐lithographed and etched silicon master was pressed onto the casted pDR1M and then put on a hot plate (55 °C temperature) for 2 h. Once the solvent was completely evaporated, the PDMS stamp was carefully removed from the Petri dish, thus leaving a pDR1M complementary structure on the glass substrate. After each use, the PDMS stamp was washed in ethanol and placed for 20 min into ultrasound bath to remove residual traces of pDR1M. Each stamp was employed three to ten times. Azopolymeric pillars had a 4 µm × 4 µm squared cross‐section and a total height of 1.3 µm. Four different pillar spacings were used: 5, 7, 9, and 11 µm, resulting in four different arrays over an area 2.5 mm × 2.5 mm each (see Figure S1, Supporting Information). In the results shown here, arrays corresponding to spatial periods 5 and 7 µm were used, which were found to induce a more significant cell response. Atomic force microscopy characterization of the patterned substrates is shown in Figure S2 in the Supporting Information.

*Optical Setup*: The setup was based on an inverted microscope (Ti‐E, Nikon) equipped with a home‐made incubator for living cells. Bright‐field imaging was performed by means of a monochromatic 16‐bit CCD camera (Apogee Ascent) placed within the eyepiece housing. From a lateral port of the microscope, a laser pattern was input and then projected onto the bottom surface of the Petri dish through the microscope objective (Nikon Plan Fluor 20×/0.50, Nikon Plan Fluor 10×/0.30). The laser pattern was generated by using an SLM illuminated by a collimated, Gaussian laser beam at 532 nm wavelength. The linearly polarized light from a doubled frequency Nd:YAG source (Torus 532, Laser Quantum) was expanded to a beam diameter of about 2 cm. The beam was directed toward the SLM (PLUTO‐VIS‐006, Holoeye Photonics AG) where a proper phase mask was displayed. The phase mask (also called Computer Generated Hologram) was calculated by applying an iterative Fourier transformation algorithm on the desired intensity pattern to project. A spatial filtering stage blocked the light component corresponding to the zeroth diffracted order, while transmitting the first diffracted order from the SLM. A lens system focused the transmitted light on the intermediate image plane, corresponding to the microscope lateral port, where the intended intensity pattern was formed. A motorized stage mounting a zero‐order half‐wave plate (Thorlabs Inc.) was used to control the orientation of the linear polarized laser pattern before projection onto the sample. By synchronizing the orientation of the half‐wave plate with the SLM, multiple patterns or pattern portions with different polarization orientations could be sequentially projected onto the sample, thus implementing complex deformed pattern with arbitrary anisotropy and inhomogeneity. In addition or in alternative, other polarization elements could be employed. For example, a polarizing device including a θ‐cell (ARCoptix S.A.)^72^ for creating beams with azimuthal, radial, or hyperbolic‐like polarization (see Movies M1–M3, Supporting Information). Following the internal optical path of the microscope, the laser intensity distribution on the intermediate image plane was projected onto the sample plane by means of the objective. The SLM could be controlled to produce an additional wave‐front curvature to the laser, thus implementing a synthetic lens with tunable focal length, for a fine matching of the laser projection system optics with the microscope internal optics. When performing observations while simultaneously illuminating the sample with the laser, a spectral filter (Semrock, Edgefilter 532) could be used in front of the CCD camera in order to block light at wavelengths below 532 nm. Irradiation times varied from few tens of seconds to 2–3 min, depending on the size of the illuminated area and the magnification factor of the microscope objective. The overall laser power reaching the sample was up to 20 mW, after losses due to i) the spatial filtering through the 10 µm pinhole in the beam expansion stage, ii) multiple reflections at tilt mirrors, and iii) diffraction at the SLM.

*Cell Culture and Manipulation*: Cell lines were cultured in Dulbecco's modified Eagle medium (MDA‐MB‐231, breast cancer cell line) or Eagle's minimal essential medium (MDCK, epithelial kidney cell line) supplemented with 10% fetal bovine serum, penicillin/streptomycin, and L‐glutamine. Cells were kept at 37°C under 5% CO_2_ humidified air. Cells were seeded on the patterned glass‐bottom Petri dish (IBIDI, #81218‐200) at a density between 2.5 × 10^4^ and 10^5^ per dish. For actin and nuclear staining, Phalloidin‐488 (Invitrogen) and either 4',6‐diamidino‐2‐phenylindole or DRAQ5 (ThermoFisher) were used. Cells were rinsed twice in phosphate‐buffered saline (PBS), fixed in 4%paraformaldehyde, rinsed again in PBS and incubated 10 min with the appropriate conjugated reagent for 10 min, rinsed and kept in PBS in a cold room prior to imaging. pLenti.PGK.LifeAct‐GFP.W and pLenti.PGK.LifeAct‐Ruby.W were gifts from R. Lansford (The Saban Research Institute, Los Angeles, CA; Addgene plasmids #51010 and #51009). LV‐GFP, RFP and CFP were a gift from Elaine Fuchs (The Rockefeller University, New York, NY; Addgene plasmids #25998; #26001; #25999).

*Microscopy*: Cells were infected with two lentiviruses carrying the transgenes LifeAct‐Ruby and H2B‐GFP which stain for polymerized actin and nuclei, respectively. Time‐lapse video‐microscopy experiments were conducted either on a Nikon Ti‐E inverted microscope (bright‐field only), or on a Leica SP8 inverted confocal microscope equipped with a motorized stage and an incubator to keep the plate stably at 37° and 5% CO_2_. Imaging on fixed cells was performed either with the confocal microscope or on an inverted widefield (Leica AF6000).

*Image Processing and Analysis*: Segmentation of nuclear and cytoplasmic signals was performed by means of Ilastik.^73^ Segmented images were then used to perform quantitative analysis with custom written MATLAB (The Mathworks) scripts. Alignment of MDA‐MB‐231 along the direction of deformation of the patterned substrate was assessed by calculating the scalar product between the direction of each cell and the local direction of deformation of each pattern. Cell orientation was defined as the direction of the major axis of the segmented cytoplasmic signal for each cell. The local direction of deformation of the substrate was calculated by creating a 2D function expressing the orientation of the polarization field in each spatial point. Alignment of single cells was then assessed by plotting the value of the cosine of the angle between the major axis of the cell and the local direction of deformation of the pattern.

Tracking of MDA‐MB‐231 was performed by segmenting nuclear signal and by implementing a tracking algorithm based on the minimum distance between objects in different frames. Segments of tracks corresponding to 1 h were used to calculate alignment onto the local direction of the deformation.

Colony growth was measured by segmenting cytoplasmic signal to obtain the outline of the colony and then calculating area and orientation of the colony at each time as the major axis of the segmented colony. Cell division orientation was measured by projecting the direction defined by the line joining the two daughter cells at each mitosis onto the local direction of deformation of the pattern.

*Data Processing and Analysis*: Violin plots were used to represent statistical distribution of the different measurements performed.^74^ Statistical tests were performed on distributions by using two‐sample Kolmogorov–Smirnov^49^ goodness‐of‐fit hypothesis tests.

## Conflict of Interest

The authors declare no conflict of interest.

## Supporting information

Supplementary

Supplementary

Supplementary

Supplementary

Supplementary

Supplementary

Supplementary

Supplementary

Supplementary

Supplementary

Supplementary

Supplementary

Supplementary

Supplementary

Supplementary
