# Supplementary material for: Driving Cells with Light‐Controlled Topographies
Source: Adv Sci (Weinh). 2019 May 20;6(14):1801826. doi: 10.1002/advs.201801826 (PMC6661947; doi:10.1002/advs.201801826)
Supplement: Supplementary file 1 — Supplementary [file ADVS-6-1801826-s002.pdf]

Copyright WILEY-VCH Verlag GmbH & Co. KGaA, 69469 Weinheim, Germany, 2019.

ADVANCED SCIENCE
Open Access

Supporting Information

for *Adv. Sci.,* DOI: 10.1002/advs.201801826

Driving Cells with Light-Controlled Topographies

Alberto Puliafito, Serena Ricciardi, Federica Pirani, Viktorie Čermochová, Luca Boarino, Natascia De Leo, Luca Primo, and Emiliano Descrovi**

Driving cell with light-controlled topographies

Supporting Information

Alberto Puliafito,[1,2,*] Serena Ricciardi,[3] Federica Pirani,[3] Viktorie Čermochová,[4,3] Luca Boarino,[5] Natascia de Leo,[5] Luca Primo,[1,2] and Emiliano Descrovi[3,†]

[1]*Candiolo Cancer Institute FPO-IRCCS, Candiolo, Turin, Italy*
[2]*Department of Oncology, University of Turin, Turin 10060, Italy*
[3]*Department of Applied Science and Technology, Polytechnic University of Turin, C.so Duca degli Abruzzi 24, Turin, 10129, Italy*
[4]*Department of Chemical Engineering, University of Chemical Technology Prague, Technická 3166 28 Praha 6, Czech Republich*
[5]*Quantum Research Labs & Nanofacility Piemonte, Nanoscience & Materials Division, Istituto Nazionale di Ricerca Metrologica, Strada delle Cacce 91, 10135 Turin, Italy*

(Dated: April 5, 2019)

SUPPLEMENTARY MOVIES LEGENDS

Movie M1-M3

Bright-field optical observation of a micro-pillar array during deformation induced by azimuthally (M1), radially (M2), hyperbolic (M3) polarized doughnut beams. The laser radiation is spectrally blocked by an edge filter located in front of the recording camera.

Movie M4

Bright-field optical observation of a micro-pillar array during deformation induced by a sequence of complementary patterns having mutually orthogonal polarization. The laser radiation is spectrally blocked by an edge filter located in front of the recording camera.

Movie M5

Inhomogeneous micropillar deformation in presence of living cell. The projected laser distribution is defined in such a way that direct illumination of the target cell is avoided.

Movie M6

Anisotropic micropillar deformation in presence of living cell. The projected laser distribution is azimuthally polarized and illuminates living cell attached on the substrate. Irradiation time is about 30 seconds, with no photoxicity effects observed.

Movie M7-M10

Time lapse obtained by monitoring MDA-MB-231 cells infected with a lentivirus carrying the H2B-CFP transgene. Green: H2B-CFP signal, Red: autofluorescence of the pattern. Each movie is relative to a representative time-series of cells migrating on azimuthal (M7), hyperbolic (M8), linear (M9) and radial (M10) deformation patterns.

* Co-corresponding author: alberto.puliafito@ircc.it
† Co-corresponding author: emiliano.descrovi@polito.it

Movie M11-12

Time lapse obtained by monitoring MDCK cells infected with two lentiviruses carrying the H2B-GFP and the LifeAct-Ruby transgenes. Green: H2B-GFP signal, Red: LifeAct-Ruby and autofluorescence of the pattern. Movie M11 is relative to growth of cells onto undeformed substrates, while movie M12 to cells growing on deformed substrates.

Movie M13

The movie shows the timelapse obtained by monitoring MDCK cells (left panel) and the quantification of cells division orientation and positioning during the course of the movie. Each couple of colored dots corresponds to the position of the daughter cells immediately after mitosis and the colored arrows the corresponding orientation. Green: H2B-GFP signal, Red: LifeAct-Ruby and autofluorescence of the pattern.

Movie M14

The movie shows the irradiation of a patterned surface, the corresponding deformation of pillars and the subsequent alignment of cells. Timeframe changes during the course of the movie in order to appreciate the relevant changes in the image.

SUPPLEMENTARY FIGURES

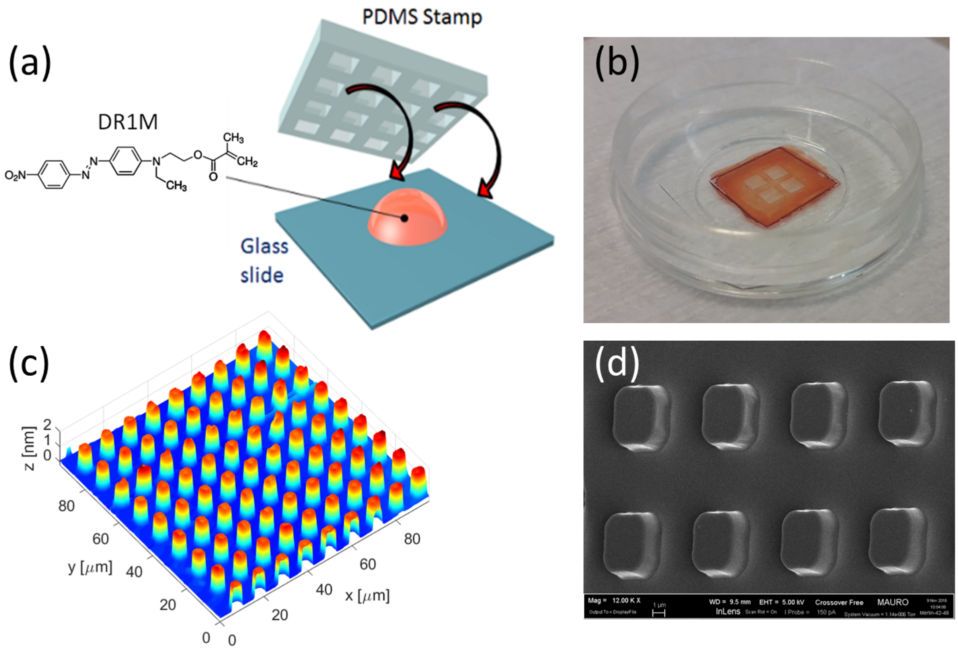

FIG. S1. (a) Sketch of the soft-printing technique used to pattern Petri dishes with arrays of micro-pillars; (b) illustrative picture of a real sample; topography of an exemplary DR1M pillar array (spacing 11 μm) as mapped by Atomic Force Microscopy (c) and Scanning Electron Microscopy (d).

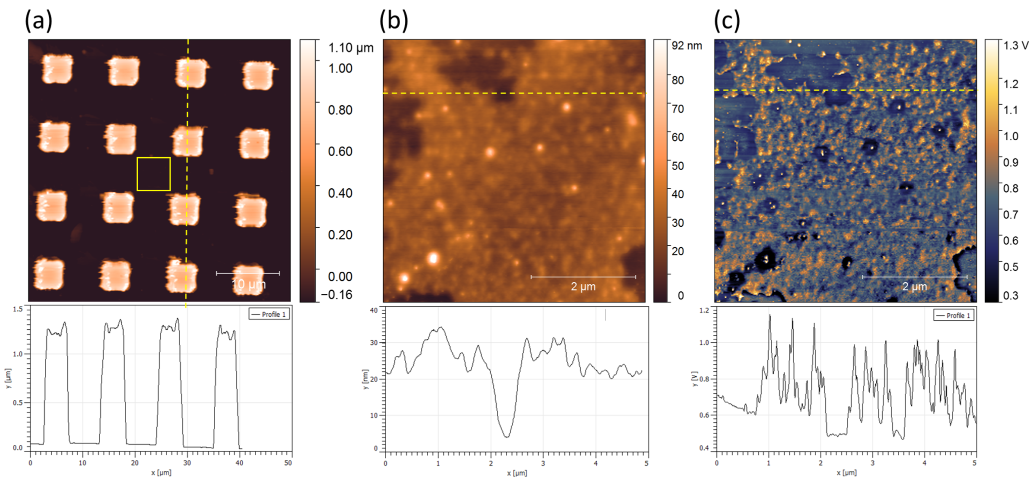

FIG. S2. (a) Tapping-mode Atomic Force Microscopy (AFM) mapping of DR1M pillars. Surface area 42μ x 42μm. In this representative samples, pillars are arranged over a squared lattice with a period of 11 μm along two orthogonal directions. (b,c) AFM topography and phase images of a flat 5 μm x 5 μm area between pillars. Both phase and topography maps reveal an inhomogeneous residual DR1M layer resulting from the soft-printing process. In particular, the phase image highlights the presence of regions with different elastic modulus that can be reasonably associated to the azopolymeric film and the glass substrate, respectively. From height discontinuities, the residual layer is estimated to be about 30 nm thick, with an average roughness of about 6 nm.

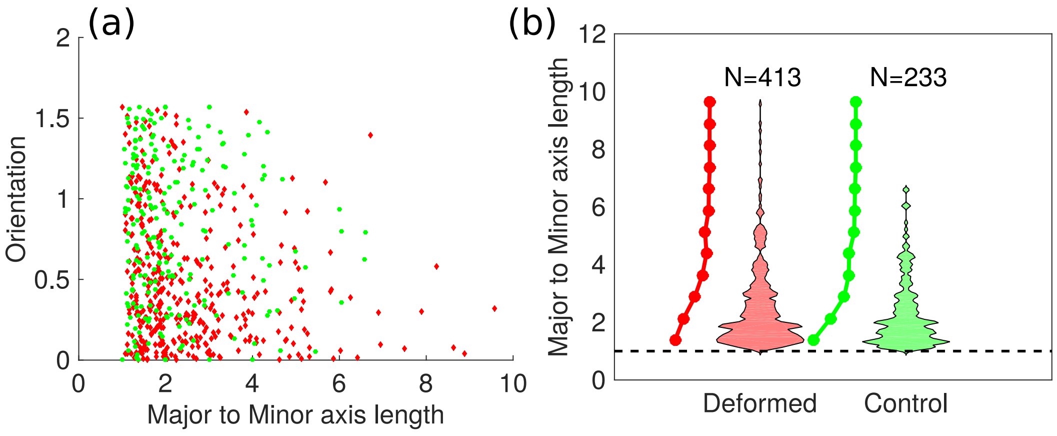

FIG. S3. (a) Quantification of the correlation between cell deformation (indicated by the ratio of Major to Minor axis of each cell) and orientation adhering on deformed (red) and non deformed (green) patterns. (b) Violin plot of cell aspect ratio adhering to deformed and non deformed substrates.

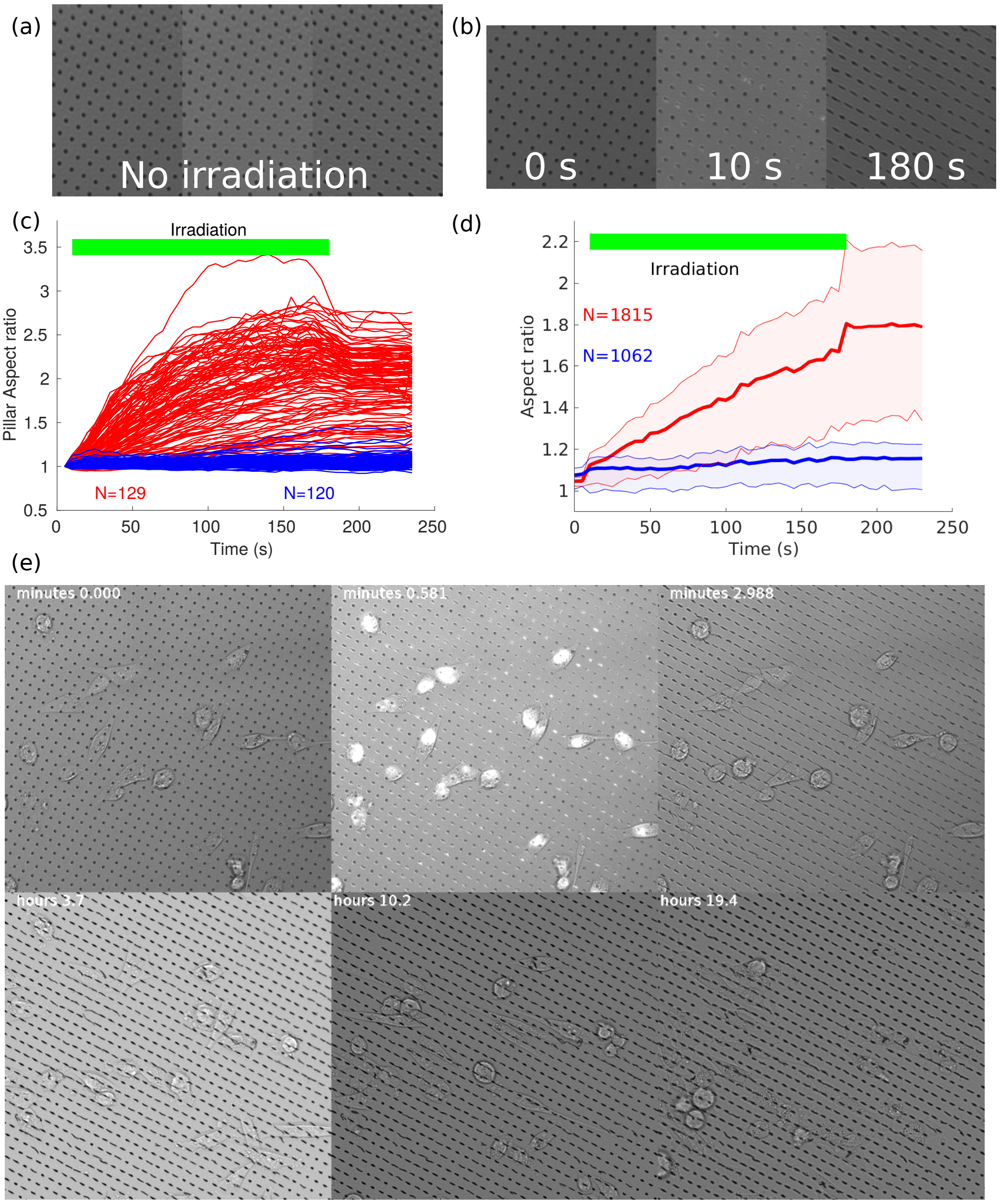

FIG. S4. (a) and (b) Snapshots of regions of the same field of view showing pillars that are not irradiated (left) or irradiated for 3 minutes with the laser. Times of the snapshots correspond to 0, 10 and 180 s. (c) Single pillars from snapshots (a) and (b) were segmented and the aspect ratio was measured. Each line represent the timetrace of the aspect ratio of one pillar. The difference between regions that have been irradiated (red) or not (blue) is evident. (d) Quantification of pillars deformation in a full field of view (see panel (e)). Thick lines represent medians and thin lines 25th and 75th quartiles respectively. Red lines correspond to irradiated regions, while blue to non irradiated regions. (e) Time sequences of cells sitting on photo-inducible substrates. The upper row of snapshots describes the irradiation. Cells fluoresce when irradiated as they are expressing H2B-RFP constructs. The lower row of snapshots illustrate the process of alignment of cells. Alignment of cells is clear after a few hours.

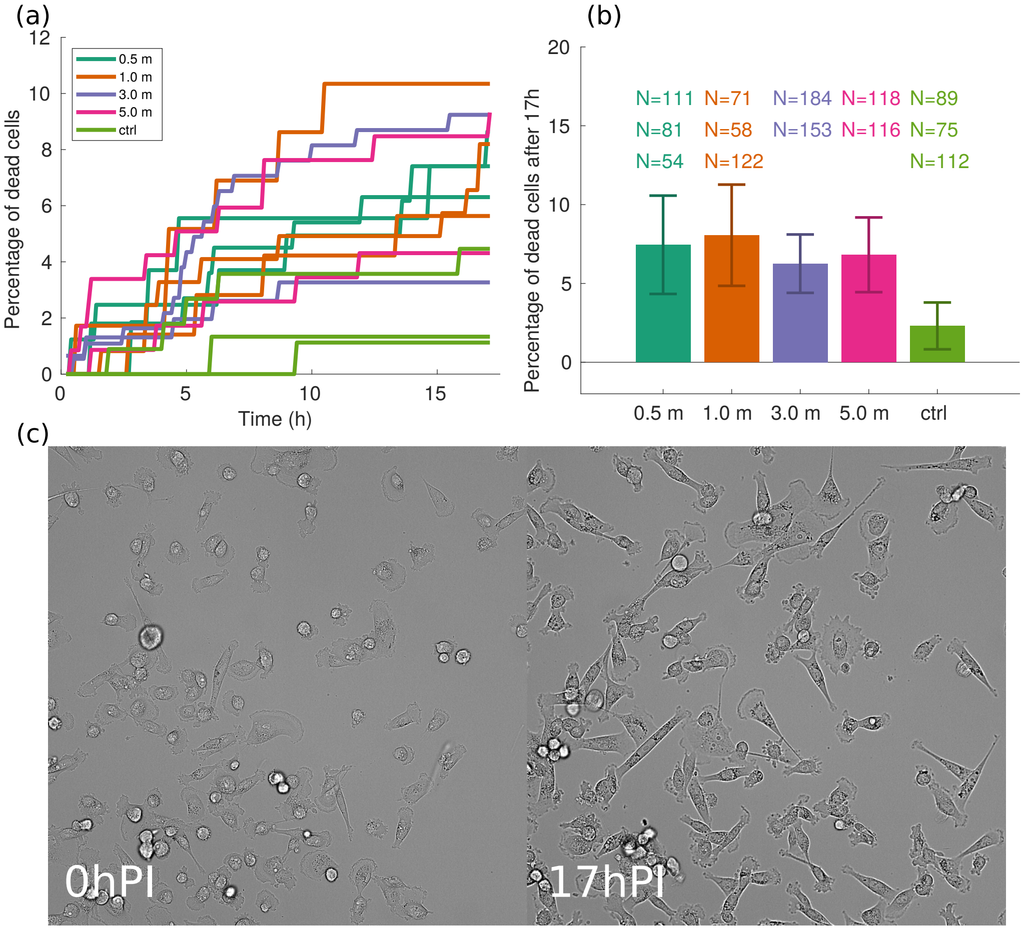

FIG. S5. (a) Quantification of cell death upon laser irradiation. Each line represent the cumulative number of apoptosis events detected by manual segmentation of bright field images acquired every 6 minute. Each color codes for an irradiation time, and different lines with the same color are replicates. The number of events is normalized with respect to the number of cells present in the first frame of the movie. (b) Comparison of cumulated events after 17h. Bar represents mean of 2 or 3 replicates, each with an initial number of cells indicated on top of the bar. Errorbars are propagated Poisson uncertainties. In our setting, phototoxicity induced by irradiation is extremely small. (c) Typical bright field snapshots as from the experiment in (a) and (b). The images shown correspond to 0 and 17 hours post-irradiation (PI), with the longest irradiation of 5 minutes.
